# The latitudinal and longitudinal allelopathic patterns of an invasive alligator weed (*Alternanthera philoxeroides*) in China

**DOI:** 10.1371/journal.pone.0280866

**Published:** 2023-01-23

**Authors:** Si-Yi Hu, Hui Gao, Jian Li, Yan-Hong Wang, An-Guo Gao, Ji-Hui Wen, Mohamed Abdelaziz Balah, Ai-Ping Wu

**Affiliations:** 1 Ecology Department, College of Resources and Environment, Hunan Provincial Key Laboratory of Rural Ecosystem Health in Dongting Lake Area, Hunan Agricultural University, Changsha, China; 2 College of Bioscience and Biotechnology, Hunan Agricultural University, Changsha, China; 3 School of Forestry and Bio-technology, Zhejiang Agriculture & Forestry University, Hangzhou, China; 4 Institute of Plant Protection, Hunan Academy of Agricultural Sciences, Changsha, China; 5 Huaihua University, Huaihua City, Hunan Province, China; 6 Ecology and Dry Lands Agriculture Division, Plant Protection Department, Desert Research Center, El Matariya, Cairo, Egypt; Universidade Federal de Alfenas, BRAZIL

## Abstract

Allelopathy has been considered a good explanation for the successful invasion of some invasive plants. However, the real latitudinal and longitudinal allelopathic effects on native species have rarely been documented since many exotics have spread widely. We conducted a Petri dish experiment to determine the latitudinal and longitudinal allelopathic patterns of an invasive alligator weed (*Alternanthera philoxeroides*) on a common crop (*Lactuca sativa*) in China, and find what determines the allelopathic intensity. The results showed that the allelopathic effects of *A*. *philoxeroides* increased with the latitude while decreased with the longitude. This indicated that *A*. *philoxeroides* used its allelopathy to gain competitive advantages more in its recent invaded communities than that in its early invaded ones as *A*. *philoxeroides* is expanding from southeast China to northwest China. Furthermore, we found that the allelopathic intensity of *A*. *philoxeroide* was negatively correlated to the leaf contents of soluble carbohydrate (SC), carbon (C) and nitrogen (N), but that was positively correlated to the leaf contents of soluble protein (SP), free amino acids (FAA), plant polyphenol (PP), phosphorus (P) and potassium (K). These results suggested that the allelopathic intensity of *A*. *philoxeroide* was more determined by the limited P and K nutrients as well as the intermediate allelochemicals (SP, FAA, PP) rather than the unlimited C, N and SC. Thus, we can speculate that the negative or positive effects of plant aqueous extracts are a function of not only the extract concentrations but also the trade-offs between inhibition and promotion of all components in the extracts. Then we could reduce the allelopathic effects of *A*. *philoxeroide* by controlling the component contents in the plant tissues, by fertilization or other managements, especially in the plant recent invaded communities.

## Introduction

A large number of invasive plants produce allelochemicals inhibiting their co-occurring native species to get competitive advantages, and the process is known as allelopathy, namely, “novel weapon hypothesis” in invasion ecology [1, 2]. Allelochemicals act directly or indirectly on plants, affecting plants’ physiological and biochemical processes through inhibition of plant germination, growth and development [3]. The suppression of other species provides invasive plants competitive advantages of resources and facilitates their colonization on new habitats [4]. The release of allelochemicals into the environment occurs naturally through rainfall leaching, evaporation and root secretion [5]. Though there are many kinds of allelochemicals, water leaching is a common method for extracting allelochemicals, most of which are water-soluble, and then allelopathic effects are usually conducted in Petri dish bioassays [6, 7]. Pile-up studies have proven that many invasive plants gain their advantages by exerting negative allelopathic effects on their co-occurring native species, these exotics include *Mikania micrantha*, *Solidago canadensis*, *Ambrosia artemisiifolia*, *Lantana camara* and so on [8–10]. The allelopathic effects of invasive plants with different plant tissues or organs (or their mixtures), residues, ages and various concentrations of raw plant materials or pure allelochemicals, and even different exposure times, have been well investigated in previous studies [7, 11, 12]. However, the plant materials in these researches are collected from a limited area rather than the whole invaded ranges of the exotics, which have expanded over large areas along latitudes and longitudes. Thus, the latitudinal and longitudinal allelopathic patterns of invasive plants are a large knowledge gap in invasion ecology although the allelopathic effects of invasive plants decrease with increasing invasion phase are frequently reported [13]. Accordingly, research in this area can advance our ecological understanding of the success of invasive plants in terms of allelopathy.

The secretion of allelochemicals is influenced by both the plant itself and other biotic and environmental factors [14]. For example, a plant allelopathic intensity is increased when the plant is unfit or under stress conditions [15]. However, the process of allelochemical excretion is energy-consuming, which would affect a growth and development of the plant due to the limited resources in the plant. Thus, plants including invasive plants have to trade-off their resources between their rapid growth and maintenance of chemical defense [16]. As far as nutrients are concerned, nitrogen (N), phosphorus (P) and potassium (K) are paid much more attention, and carbon is neglected [16, 17], when allelopathy is linked to resources, although most allelopathicals are carbonaceous [5]. Furthermore, which element determines the allelopathic intensity of a plant has not been documented by our best knowledge. Accordingly, we can manage the allelopathic effect of an invasive plant by controlling or adjusting the nutrient supplies if we know which nutrient is responsive for this effect.

*Alternanthera philoxeroides* is a plant of the Amaranthus family native to Brazil, it is highly invasive and has developed into a globally pernicious weed [18]. Since its introduction to China in the 1930s as a forage crop in Southern China, it has gradually expanded from the southeast coast to more than twenty provinces and was included in the First List of Invasive Alien Species in China in 2003 [19]. There are two types of *A*. *philoxeroides*, aquatic and terrestrial ecotypes [20], with a wide range of adaptations and obvious competitive advantages. It is found in various habitats such as lawns, agricultural fields, river (water) banks, and wastelands, and has caused great harm to agriculture, horticulture, farming, and transportation, and has caused serious imbalances in invaded ecosystems [21]. Allelopathic effects are considered to be an important mechanism for the successful invasion of this alien species [1]. In addition, the allelopathic effects of *A*. *philoxeroides* can vary under different nutritional conditions [22]. The strong allelopathy and extended latitudinal and longitudinal invaded ranges provide a good opportunity to conduct our analysis.

Based on the results of previous studies, we would like to further clarify the variation in the allelopathic effects of *A*. *philoxeroides* over a large latitudinal and longitudinal area. Therefore, we assessed the allelopathic effects of aqueous extracts of *A*. *philoxeroides* at different latitudes and longitudes on the germination and growth of *Lactuca sativa*. Furthermore, we want to find which element was related to the allelopathic intensity of this invasive plant. Thus, we aim to determine: (i) the real latitudinal and longitudinal patterns of allelopathic effects of this invasive plant; (ii) which element accounts for the allelopathic intensity of this invasive plant.

## Materials & methods

### Extract preparation of *A*. *philoxeroides*

From July to August in 2019, we selected 19 sites along the whole latitudinal and 26 sites along the whole longitudinal range of *A*. *philoxeroides* in China every other latitude or longitude degree, respectively (Table 1). Accounting for spatial heterogeneity, we collected plant leaves at three or four alligator weed communities (at least 1 km apart) for each of the 45 sites. In each community, we selected 50 mature and healthy leaves from 25 invasive alligator weed individuals or genets, as alligator weed has opposite leaves. The leave samples were separated into two parts, one part was used for aqueous extract preparation and the another part was used for leaf index measurements.

**Table 1:** The sample sites of *A*. *philoxeroides* along the latitudinal and longitudinal gradients in China. The oldest community of *A*. *philoxeroides* is in Shanghai city [23].

Sampling sites	Longitudinal sites	Latitudinal sites
1	122°18′E, 29°59′N	110°35′E, 20°07′N
2	121°05′E, 29°97′N	113°05′E, 22°31′N
3	120°17′E, 30°08′N	113°26′E, 23°25′N
4	119°11′E, 30°16′N	113°50′E, 24°24′N
5	118°15′E, 30°26′N	113°02′E, 25°17′N
6	117°08′E, 30°18′N	112°52′E, 26°13′N
7	115°98′E, 30°07′N	112°52′E, 27°15′N
8	115°40′E, 30°17′N	113°03′E, 28°06′N
9	114°32′E, 30°43′N	113°23′E, 29°20′N
10	113°16′E, 30°23′N	114°20′E, 30°26′N
11	112°14′E, 30°00′N	114°32′E, 30°43′N
12	111°29′E, 30°16′N	114°04′E, 31°21′N
13	110°19′E, 30°36′N	114°09′E, 32°31′N
14	109°31′E, 30°17′N	114°06′E, 33°21′N
15	108°11′E, 30°01′N	113°50′E, 34°21′N
16	107°28′E, 29°48′N	113°54′E, 35°18′N
17	106°12′E, 29°45′N	114°24′E, 36°02′N
18	105°24′E, 30°04′N	114°25′E, 36°24′N
19	104°36′E, 30°23′N	115°34′E, 38°52′N
20	103°06′E, 30°04′N	
21	102°13′E, 29°55′N	
22	102°10′E, 27°44′N	
23	101°29′E, 27°25′N	
24	100°46′E, 27°12′N	
25	99°18′E, 28°13′N	
26	98°36′E, 29°05′N	

After air drying, plant samples were processed into fine powders by milling with a mortar and a pestle. Leaf extracts were prepared by mixing 5 g of fine powder in 1 L distilled water at room temperature (25±3°C) with occasional stirring. The extract was strained through four layers of cheesecloth to remove solid materials after extracting for 24 h. The pH of aqueous extract was adjusted to 7.0 with 1 M NaOH or HCl. After that, we obtained an aqueous extract with a concentration of 5 g/L, which is a concentration commonly used in allelopathic experiments and then all extracts were kept at 4°C till use [24].

### Measurements of leaf indices

Plant samples were oven dried at 65°C for 72 h after drying at 105°C for 30 minutes, and then processed into fine powder by grinding and passing through a 0.45 μm mesh. The total N and C contents (% of dry mass) of the leaves were determined using an organic elemental analyzer vario MACRO cube-CNHS (Elementar, Germany), and the total P (% of dry mass) contents were analyzed by the vanadium molybdenum yellow colorimetric method after being digested in H_2_SO_4_ and H_2_O_2_, standardized against known reference materials. The total K contents (% of dry mass) of the leaves were determined using flame spectrophotometry after digestion with concentrated sulfuric acid and hydrogen peroxide. About 30 mg of the leaf powder was extracted with 10 ml 80% ethanol at 80°C. After centrifugation, the supernatant was used for free amino acid (FAA) and soluble carbohydrate (SC) determination. The FAA was determined by ninhydrin colorimetry method using alanine as a stand [25]. The SC was determined by phenol method using glucose as a standard [26]. After that, the soluble protein (SP) was determined using Coomassie brilliant blue G-250 with bovine serum albumin as a stand [27] and the plant polyphenol (PP) was determined by Folin-Ciocalteu colorimetric method [28].

### Preparation of target seed

Seeds of *L*. *sativa* were purchased from Hunan Academy of Agricultural Sciences. The target seeds were immersed in 5% hydrogen peroxide for 30 min to exclude other possible effects caused by bacteria and fungi, then the seeds were rinsed with enough distilled water for several times. The sterilized seeds were soaked in distilled water for 2 hours to prompt germination.

### Allelopathic experiment

Leaf aqueous extracts of *A*. *philoxeroides* from the above different sites were used in this experiment, and another control treatment with distilled water (CK) was also set. In each treatment, three replicates were used. In each replicate, 30 sterile seeds were placed in a separate Petri dish lined with two 5-cm pieces of filter paper, and 5 mL of due extract were added. All Petri dishes (138) were placed in an incubator at 14 h light (20°C) and 10 h in darkness (18°C) and 60% relative humidity for germination. When the radicle length was over 2 mm, seeds were considered germinated. The day when the first seed germinated in each dish was considered the initial germination time (IGT) and the germination was recorded daily. Germination speed (GS) was calculated as the following forum:

$$S=(N_{1}\times 1)+(N_{2}-N_{1})\times \frac{1}{2}+(N_{3}-N_{2})\times \frac{1}{3}+\ldots +(N_{n}-N_{n-1})\times \frac{1}{n}$$

N_n_ represents the quantity of germination seeds in day n.

The experiment was terminated when no seeds germinated for three consecutive days. At the end of the experiment, ten of the best grown seedlings were selected from each Petri dish. The whole leaf area (LA) of every seedling was analyzed by an analysis software (WinFOLIA 2004a, Regent Instruments Inc., Qúebec, Canadamachine) when all leaves of every seedling were scanned by a scanner (Epson Perfection 4870 Photo). Then the plant was separated into root and shoot, and the shoot height (SH) and root length (RL) were measured. The root:shoot ratio (R/L) was calculated as the RL divided by the SH.

### Data analysis

In this study, all statistical analyses were conducted using the software package R 3.5.2 [29]. Differences in IGT, germination rate (GR), GS, SH, RL, R/S and LA of the target species between the two treatments were compared by an independent sample t-test when all indices were expressed as a percentage of the control. Furthermore, linear fitting was used to indicate the relationships between all the above measured characteristics and the latitude or the longitude. Similarly, the relationships between the invasive plant leaf parameters and the indices of the target species were also analyzed by linear fitting to determine which nutrient would affect the seed germination and seedling growth of *L*. *sativa*. To compare the spatial variance in overall allelopathic effects along the latitudinal and longitudinal gradients, the seven measured indices were analyzed by principal component analysis (PCA), and the eigenvalues of the PCA axis 1 were correlated to the latitude and longitude with the best model by using the Akaike’s information criterion (AIC) procedure.

As the RL and GR of *L*. *sativa* were significantly correlated to most of the leaf parameters of the invasive *A*. *philoxeroides* (Fig 3), we only took RL and GR in this analysis. To determine how latitude impact on RL and GR of *L*. *sativa*, we conducted a generalized multilevel path models (using piecewiseSEM package) after the data were log transformed [30]. By this analysis, we can discriminate the direct and indirect effects of the latitude gradient on the invasive plant leaf parameters, which were independent of each other. In the final accepted path model, only significant paths were included and all variables that varied with latitude or impacted the R/S or GR of *L*. *sativa* were showed. The standardized path coefficients demonstrated the indirect, direct and total effects of the predictors [30].

## Results

### Allelopathic effects on *L*. *sativa*

**Fig 1:**
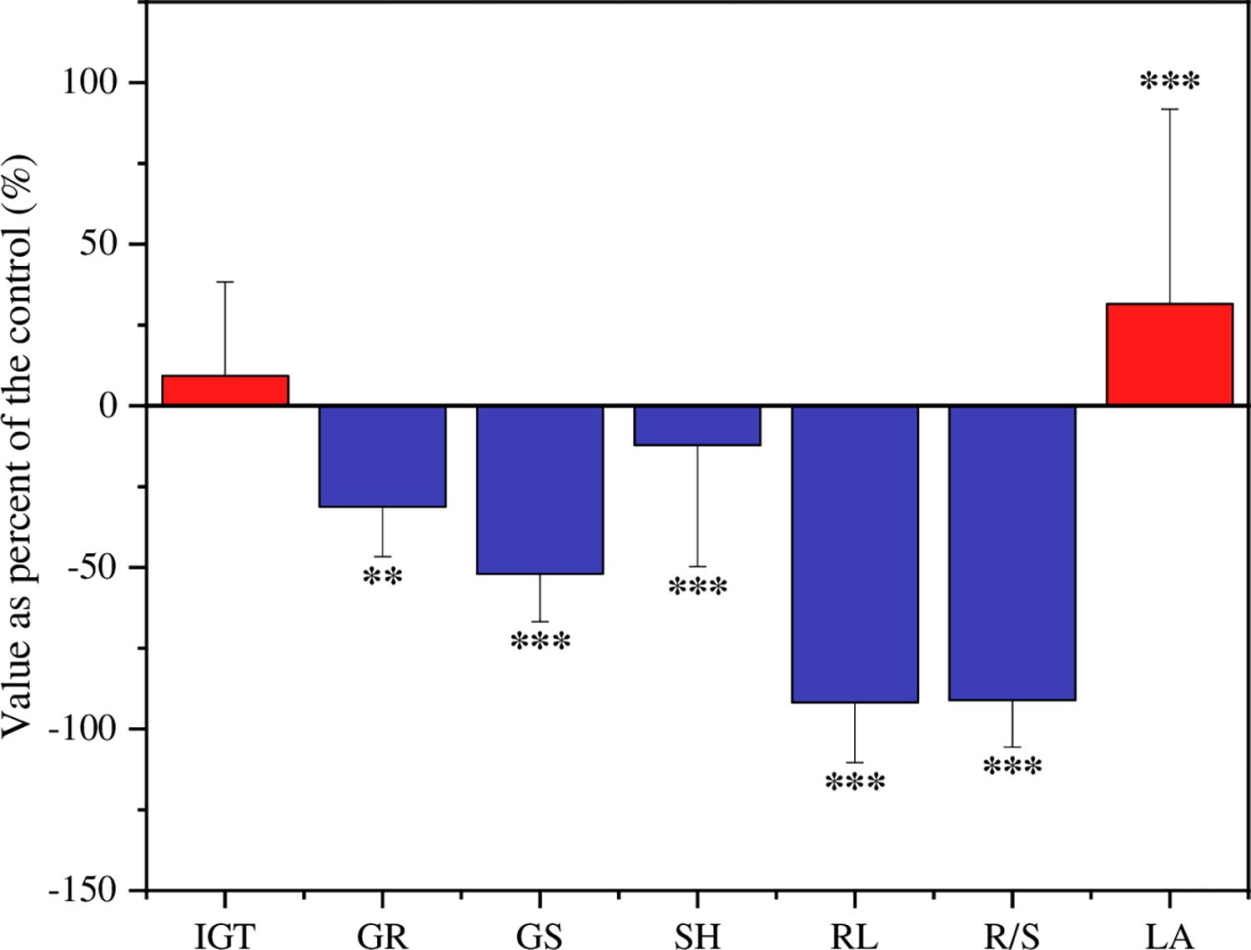
The seed germination and seedling growth indices of *L*. *sativa* treated with *A*. *philoxeroides* aqueous extracts. IGT, GR, GS, SH, RL, R/S and LA represent initial germination time, germination rate, germination speed, shoot height, root length, root length:shoot height ratio and leaf area, respectively. ‘*’, ‘**’ and ‘***’ signify *p* < 0.05, *p* < 0.01 and *p* < 0.001 compared with the control, respectively.

Generally, the seed germination and seedling growth of *L*. *sativa* were significantly impacted by the aqueous extracts of *A*. *philoxeroides* from all of the sampling sites (Table 2 and Fig 1). Namely, germination rate (GR), germination speed (GS), shoot height (SH), root length (RL) and root length: shoot height (R/S) of *L*. *sativa* were significantly inhibited by the aqueous extracts of *A*. *philoxeroides*, while leaf area (LA) and initial germination time (IGT) of *L*. *sativa* were distinctly promoted or not obviously affected by the aqueous extracts of *A*. *philoxeroides*, respectively. Furthermore, it is clear that the RL and R/S of *L*. *sativa* were the most seriously influenced by the aqueous extracts of *A*. *philoxeroides* (Fig 1) among all of the measured parameters.

**Table 2:** The seed germination and seedling growth indices of *L*. *sativa* treated with *A*. *philoxeroides* aqueous extracts from the 45 sites. IGT, GR, GS, SH, RL, R/S and LA represent initial germination time, germination rate, germination speed, shoot height, root length, root length:shoot height ratio and leaf area, respectively. ‘*’, ‘**’, ‘***’ and ‘/’ signify *p* < 0.05, *p* < 0.01, *p* < 0.001 and no significant effects compared with the control, respectively; bold letters means positive effects.

		IGT	GR	GS	SH	RL	R/S	LA
longitude (°E)	98.36	/	*	**	**	***	***	/
	99.18	/	**	***	***	***	***	**
	100.46	/	*	***	***	***	***	***
	102.10	/	**	***	***	***	***	**
	102.13	/	**	***	**	***	***	/
	103.06	/	/	**	/	***	***	**
	104.36	/	*	**	**	***	***	/
	105.24	/	*	***	*	***	***	***
	106.12	/	*	***	*	***	***	**
	107.28	/	*	**	/	***	***	***
	108.11	/	***	**	***	***	***	**
	109.31	/	/	**	/	***	***	**
	110.19	/	/	**	**	***	***	*
	111.29	/	*	**	/	***	***	***
	112.14	/	**	**	/	***	***	/
	113.16	/	/	**	*	***	***	**
	114.32	/	*	**	***	***	***	/
	115.40	/	/	**	/	***	***	*
	115.98	/	*	**	***	***	***	***
	117.08	/	***	**	/	***	***	***
	118.15	/	*	**	/	***	***	***
	119.11	/	/	**	*	/	***	***
	120.17	/	/	*	***	***	***	/
	121.05	/	*	**	***	***	***	***
	122.18	/	*	**	***	***	***	/
latitude (°N)	20.10	/	*	***	**	***	***	**
	22.51	/	/	**	/	/	/	/
	23.42	/	*	**	***	***	***	**
	24.39	/	**	***	***	***	***	/
	25.28	/	/	/	***	***	***	***
	26.22	/	*	**	*	***	***	***
	27.26	/	*	**	*	***	***	/
	28.11	/	/	**	**	***	***	***
	29.33	/	**	**	***	***	***	**
	30.44	/	*	**	***	***	***	/
	31.35	/	**	***	***	***	***	/
	32.34	/	/	/	/	***	***	*
	32.52	/	*	**	***	***	***	/
	34.25	/	*	*	/	***	***	**
	35.31	/	/	/	***	***	***	/
	36.03	/	/	*	*	***	***	/
	36.40	/	**	***	***	***	***	***
	38.87	/	*	***	*	***	***	*

### Longitudinal and latitudinal allelopathic patterns

**Fig 2:**
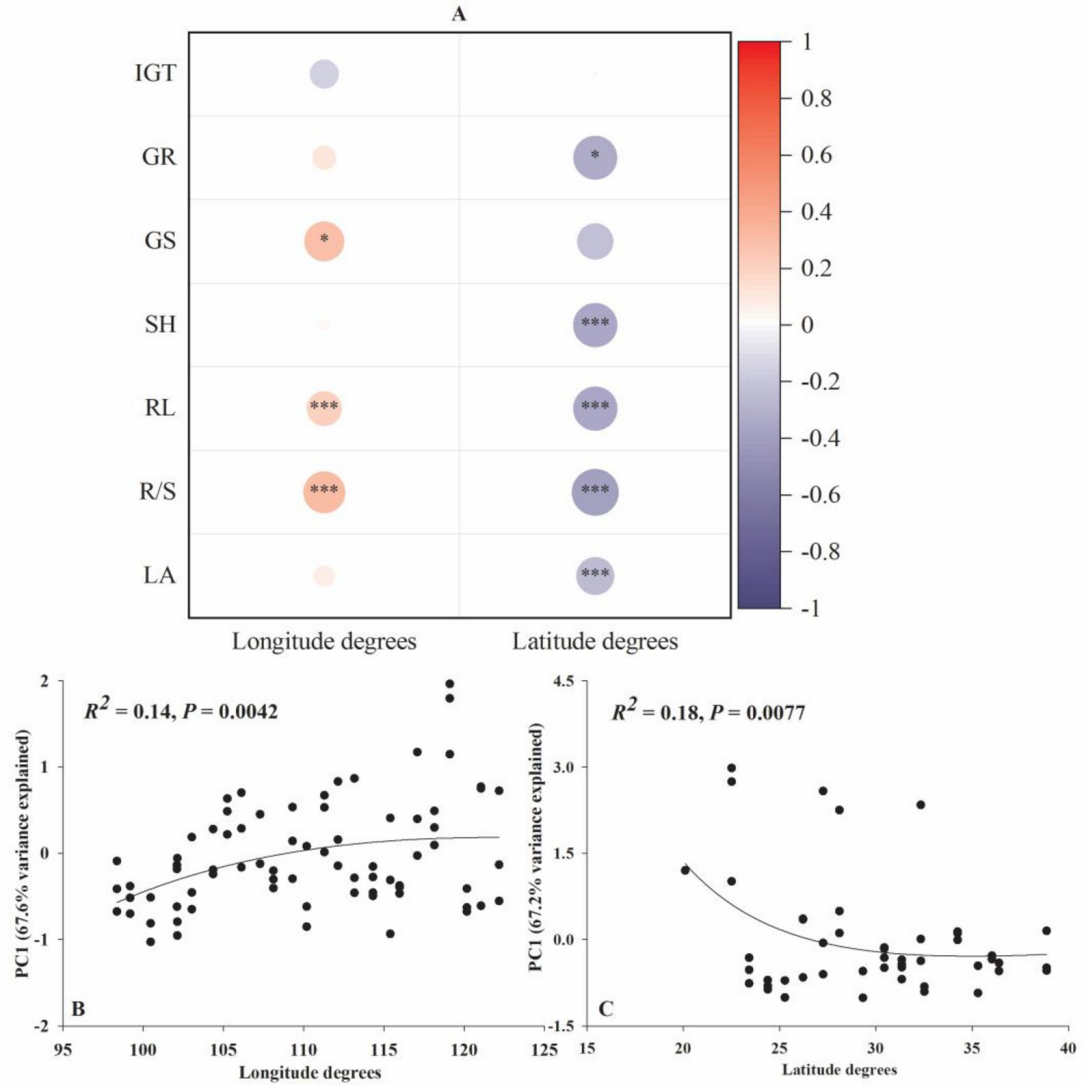
The relationships between the seed germination and seedling growth indices (A) and the PC1 eigenvalues of these indices (B and C) of *L*. *sativa* along the latitude and longitude degrees. IGT, GR, GS, SH, RL, R/S and LA represent initial germination time, germination rate, germination speed, shoot height, root length, root length:shoot height ratio and leaf area, respectively. ‘*’, ‘**’ and ‘***’ signify *p* < 0.05, *p* < 0.01 and *p* < 0.001, respectively.

Along the longitude, the PC1 (67.6% variance explained) was mainly contributed by RL and R/S, similarly, along the latitude, the PC1 (67.2% variance explained) was mainly contributed by RL, SH and R/S (S1 Table). In general, the seed germination and seedling growth of *L*. *sativa* were markedly positively correlated with the longitude degree and negatively correlated with the latitude degree (Fig 2). Along the longitude gradient, only GS, RL and R/S of *L*. *sativa* were eminently positively correlated with the longitude degree and the other indices of *L*. *sativa* were not significantly correlated with the longitude degree (Fig 2A). Adversely, along the latitude gradient, GR, SH, RL, R/S and LA of *L*. *sativa* were obviously negatively correlated with the longitude degree and the other indices of *L*. *sativa* were not significantly correlated with the longitude degree (Fig 2A).

### Allelopathic intensity determined by extract components

**Fig 3:**
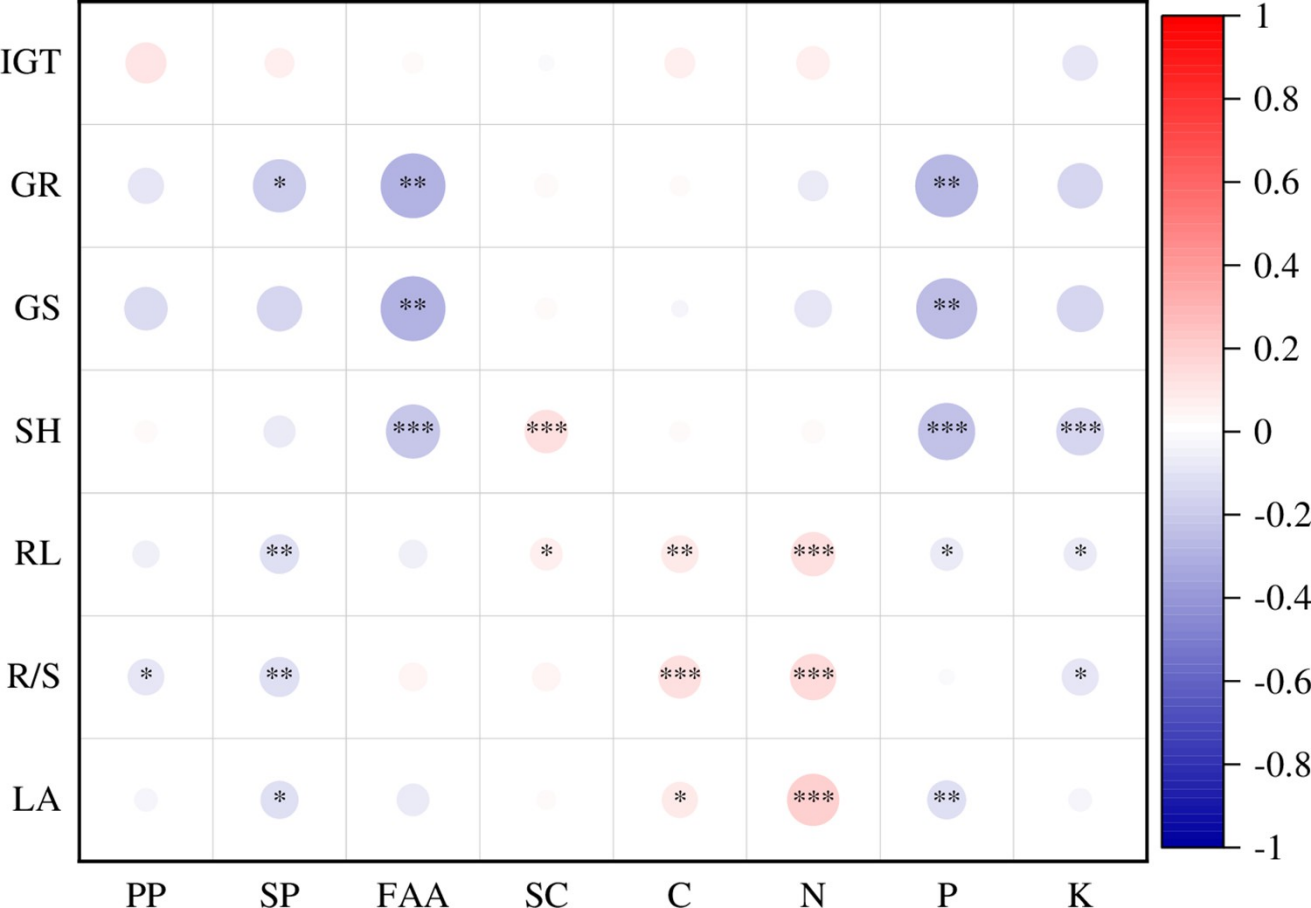
The relationships between seed germination and seedling growth indices of *L*. *sativa* and extract components of *A*. *philoxeroides*. PP, SP, FAA, SC, C, N, P and K represent plant polyphenol, soluble protein, free amino acids, soluble carbohydrate, carbon, nitrogen, phosphorus and potassium. ‘*’, ‘**’ and ‘***’ signify p < 0.05, p < 0.01 and p < 0.001, respectively.

Totally, seed germination of *L*. *sativa* was negatively correlated with the plant leaf indices of *A*. *philoxeroides* while seedling growth of *L*. *sativa* was negatively or positively correlated to the plant leaf indices of the invasive *A*. *philoxeroides* (Fig 3). Namely, GR and GS of *L*. *sativa* were negatively correlated to the plant leaf contents of SP, FAA and P of the invasive *A*. *philoxeroides* and the other relationships between seed germination parameters of *L*. *sativa* and the plant leaf indices of the invasive *A*. *philoxeroides* were not significant (Fig 3). Similarly, the SH of *L*. *sativa* was negatively correlated to the plant leaf contents of FAA, P and K of the invasive *A*. *philoxeroides*. Furthermore, the RL, R/S and LA of *L*. *sativa* were also negatively correlated to the plant leaf contents of SP, P and K of the invasive *A*. *philoxeroides*. On the contrary, RL, R/S and LA of *L*. *sativa* were positively correlated to the plant leaf C and N contents of the invasive *A*. *philoxeroides*. Moreover, the plant leaf FAA contents of the invasive *A*. *philoxeroides* was also positively correlated to the SH and RL of *L*. *sativa* (Fig 3).

### Path model analysis

**Fig 4:**
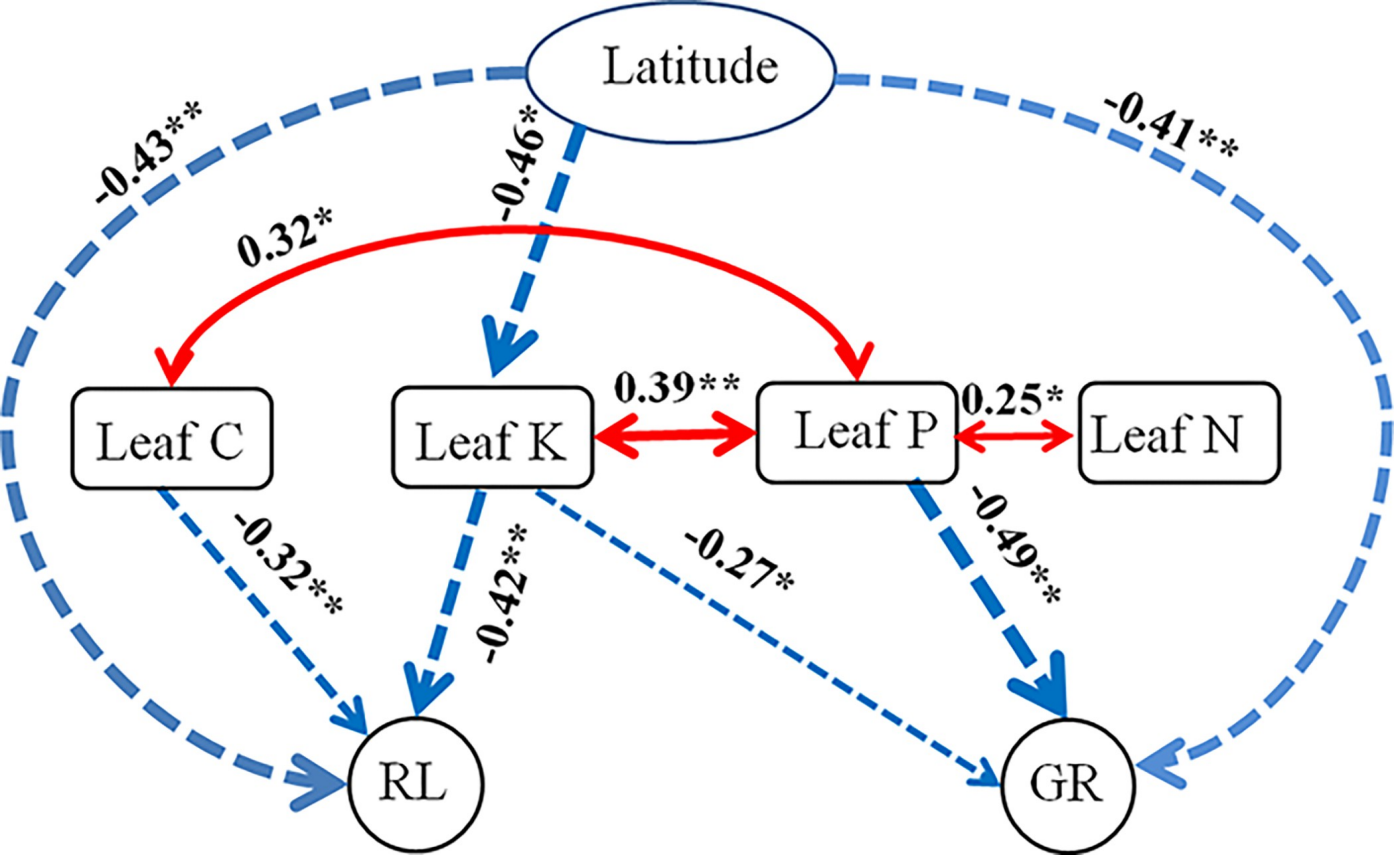
Results of the generalized multilevel path models: Latitude effects plant leaf potassium content (plant K), root length (RL) and germination rate (GR) of the target species; plant K content on RL and GR of the target species; plant leaf carbon content (plant C) on RL of the target species; plant leaf phosphorus content (plant P) on GR of the target species. Only significant paths are presented. Dashed arrows signify negative effects and solid arrows represent positive effects. The width of the arrows denotes the magnitude of standardized path coefficients. ‘*’, ‘**’ and ‘***’ signify *p* < 0.05, *p* < 0.01 and *p* < 0.001, respectively.

Our accepted path model suggested that the latitude increase could directly impacted or indirectly affected through changes in plant K content on the RL and GR of *L*. *sativa* (Fig 4). While the plant C content had a direct effect on RL of *L*. *sativa* and the plant P content also had a direct effects on GR of *L*. *sativa*. This path analysis implied that the plant K content decreased with the latitude, and both of the plant K content and the latitude negatively influenced the RL and GR of performance *L*. *sativa*. Furthermore, both plant C and P contents negatively affected the RL and GR of *L*. *sativa*, respectively.

## Discussion

### Allelopathic effects on *L*. *sativa*

Agreed with many former studies, the invasive aggressive weed *A*. *philoxeroides* showed a strong allelopathic effect on the seed germination and seedling growth of *L*. *sativa* [3, 31]. This might beneficiate the invasion success of the invasive plant although other well-explained mechanism about its super competitive ability has been reported [32–34]. For all of the studied indices of *L*. *sativa*, only IGT was not affected by the aqueous extract of alligator weed, which indicates that IGT of *L*. *sativa* is not sensitive as the other characteristics. Compared with the control, the RL and R/L were the most negatively impacted, the results suggest that the roots are more affected by allelopathic effect than the above-ground parts are, as roots are directly exposed to the aqueous extracts and can absorb allelochemicals from the solution directly [24]. However, LA of *L*. *sativa* was promoted by the aqueous extract of alligator weed. This might because that the LA of *L*. *sativa* is promoted by the low concentration of the aqueous extracts, in accord with former studies [35]. Overall, the different responses of the determined characteristics of the target species to the allelopathy is clearly due to their different sensitivity or the index specificity within a species [36–38]. In this study, our results showed that the leaves of *A*. *philoxeroides* from all of the sampling sites demonstrated strong allelopathic effects on the seed germination and seedling growth of the target species (Table 2). The allelopathy of an invasive plant from its broad latitudinal and longitudinal invaded range were first reported in this study, comparing to a narrow limited area in most previous research [3, 6, 8, 9, 11, 31].

### Allelopathic effects along longitude and latitude

Our results indicated that the allelopathhic effects of *A*. *philoxeroides* on the target species increased along the latitude degree but decreased with the longitude degree. This implies that the allelopathy strength of the alligator weed decreased with the plant invasion stages as *A*. *philoxeroides* is reported to spread from the southeast China to the northwest China [23, 39]. Adversely, in the early invaded communities, invasive plants tend to gain higher growth rates to yield more biomass and may reduce inputs to the production of allelochemicals responding to the intraspecific competition increase [13]. In the recently invaded communities, invasive plants have to allocate more resource to secrete allelochemicals to inhibit the growth of other plants responding to the interspecific competition increase [15]. Similarly, Lankau et al. found a significant decrease in phytotoxin production of *Alliaria petiolata* from early invaded to recently invaded populations, with a concomitant decrease in its impact on other plants [40–42].

A plant, usually, allocates more resources to growth and development and less nutrients to chemical defense when it is under favourable environmental conditions [43]. In contrast, a plant produces more allelochemicals as a plant defense mechanism to obtain higher levels of yield [15, 16], when it grows in unfavourable environmental conditions [44]. Accordingly, a plant has to trade-off its growth and defense according to the habitat it grows [45]. In China, the environment in the north and in the west is more stressful (colder, drier or higher altitude) than that in the south and in the east [46]. The relatively harsher environments in the northern and western China might stimulate the allelochemical exudation of the alligator weed. Thus, the allelopathic intensity of the alligator weed is greater in the northern and western China than that in the southern and eastern China. This trend well interprets the latitudinal and longitudinal allelopathic pattern of the alligator weed in China at least from the perspective of plant growth and defense trade-offs.

### Allelopathic effects with extract components

It is visible that the seed germination of the target plant was more sensitive than seedling growth to the aqueous extract components of the alligator weed from the results in Fig 1. This result is similar to the responses of twenty eight plants to the allelopathy of *Mikania micrantha* [6, 24]. The reason might because that the seeds were more directly and earlier affected by the aqueous extracts. The R/S of *L*. *sativa* was negatively correlated to the PP of the alligator weed, which is because that phenolics play an important role in plant allelopathic potential [47, 48]. Usually, proteins and amino acids are involved in various physiological and biochemical reactions in plants; moreover, they play an important role of secondary metabolism or directly act as allelochemicals [49, 50]. Accordingly, the seed germination and seedling growth of *L*. *sativa* were negatively affected by the FAA and SP of the alligator weed. However, the negative relationships between seed germination and seedling growth of *L*. *sativa* and the P and K of the alligator weed might because the P limitation is pervasive in terrestrial ecosystem [51] and K is accumulated by the alligator weed [52]. Furthermore, K can enhance plant tolerance to different harsh environments, strengthen plant defense and alleviate environmental stress [53–55]. Our accepted path model also strongly supports the phenomenon that K is more important than P in term of the allelopathy of the alligator weed in this study (Fig 3). However, the seed germination and seedling growth of *L*. *sativa* were promoted by the contents of SC, C and N of *L*. *sativa*, this might because these elements are not involved in allelopathy or their concentrations are too low to take effect [56]. Thus, they could be used as resources in the plant seed germination and seedling growth. Moreover, we can speculate that the negative or positive effects of plant aqueous extracts are a function of not only the extract concentrations [6, 11] but also the trade-offs between inhibition and promotion of all components in the extracts although we did not conduct different extract concentration experiment in this study. However, this deserves further research.

## Conclusion

Our results confirmed the strong allelopathic effects of the aqueous extract of *A*. *philoxeroides* on the seed germination and seedling growth of *L*. *sativa* in a broad latitudinal and longitudinal invaded range. Furthermore, the allelopathic effects of *A*. *philoxeroides* increased with the latitude while decreased with the longitude. Moreover, the results suggested that the allelopathic intensity of *A*. *philoxeroide* was more determined by the limited P and K nutrients as well as the intermediate allelochemicals (SP, FAA, PP) rather than the unlimited C, N and SC. The study indicates that we could reduce the allelopathic effects of *A*. *philoxeroide* by controlling the component contents in the plant tissues, by fertilization or other managements, especially in the plant recent invaded communities.

## Supporting information

S1 Table The component score coefficient matrix of the seven measured indices along the longitude and latitude by principal component analysis. (DOC)

S1 Data (XLS)
