# Supplementary material for: The latitudinal and longitudinal allelopathic patterns of an invasive alligator weed (Alternanthera philoxeroides) in China
Source: PLoS One. 2023 Jan 23;18(1):e0280866. doi: 10.1371/journal.pone.0280866 (PMC9870113; doi:10.1371/journal.pone.0280866)
Supplement: S1 Table — (DOC) [file pone.0280866.s001.doc]

Supporting table 1 The Component Score Coefficient Matrix of the seven measured indices along the longitude and latitude by principal component analysis.

| Measured indices | Longitude Component Latitude Component | | | |
| --- | --- | --- | --- | --- |
|  | 1 2 1 2 | | | |
| IGT | 0.019 | -0.059 | 0.006 | -0.310 |
| GR | 0.013 | -0.055 | 0.018 | 0.005 |
| GS | 0.012 | -0.038 | 0.018 | 0.106 |
| SH | 0.118 | 0.389 | 0.198 | -0.181 |
| RL | 0.645 | -0.152 | 0.614 | -0.317 |
| RS | 0.256 | -0.420 | 0.177 | 0.004 |
| LA | 0.095 | 0.749 | 0.117 | 0.879 |

Note: IGT, GR, GS, SH, RL, R/S and LA represent initial germination time, germination rate, germination speed, shoot height, root length, root length:shoot height ratio and leaf area, respectively.
